# The regulatory role of exosomes in venous thromboembolism

**DOI:** 10.3389/fcell.2022.956880

**Published:** 2022-08-24

**Authors:** Sheng-Lin Ye, Wen-Dong Li, Wei-Xiao Li, Lun Xiao, Feng Ran, Meng-Meng Chen, Xiao-Qiang Li, Li-Li Sun

**Affiliations:** ^1^ Department of Vascular Surgery, Nanjing Drum Tower Hospital, The Affiliated Hospital of Nanjing University Medical School, Nanjing, China; ^2^ Department of Vascular Surgery, Henan Provincial People’s Hospital, Zhengzhou, China; ^3^ School of Electronic Engineering, Nanjing Xiaozhuang University, Nanjing, China

**Keywords:** exosomes, thrombosis, venous thromboembolism, biomarker, therapeutic vector

## Abstract

Exosomes are nanoscale endocytic vesicles, 30–150 nm in diameter, secreted by most cells. They mainly originate from multivesicular bodies formed by intracellular invagination of lysosomal microparticles, and released into the extracellular matrix after fusion of multivesicular bodies with cell membrane. Studies have shown that exosomes contain a variety of active molecules, such as proteins, lipids and RNAs (such as mRNA, miRNA, lncRNA, circRNA, etc.), which regulate the behavior of recipient cells and serve as circulating biomarkers of diseases, including thrombosis. Therefore, exosome research is important for the diagnosis, treatment, therapeutic monitoring, and prognosis of thrombosis in that it can reveal the counts, surface marker expression, protein, and miRNA cargo involved. Recent studies have shown that exosomes can be used as therapeutic vectors for tissue regeneration and as alternative vectors for drug delivery. In this review, we summarize the physiological and biochemical characteristics, isolation, and identification of exosomes. Moreover, we focus on the role of exosomes in thrombosis, specifically venous thromboembolism, and their potential clinical applications, including as biomarkers and therapeutic vectors for thrombosis.

## 1 Introduction

Venous thromboembolism (VTE) develops because of disturbed blood flow or stasis, hypercoagulation, or endothelial dysfunction due to vessel injury or inflammation. The main forms of VTE are deep vein thrombosis (DVT) and pulmonary embolism. The annual fatality rate of definite or probable VTE has been estimated at 23% (Tagalakis et al., 2013). Approximately 1–2 of every 1000 adults worldwide are diagnosed with VTE annually (Silverstein et al., 1998; Day, 2014). However, the underlying mechanism is not completely understood.

Cell membrane-derived extracellular vehicles (EV) originate in various cell types, including platelets, endothelial cells, erythrocytes, and leukocytes, and can be detected in human body fluids. EVs can be divided into three major groups based on their biological origin, size, and major protein markers: exosomes, macrovesicles (MVs), and apoptotic bodies. Researchers are paying increasing attention to the precise biological roles of these vesicles in different processes in the human body (van der Pol et al., 2012; Colombo et al., 2014; Hromada et al., 2017). Over the past few years, membrane-derived EVs have been recognized as important molecules for intercellular communication in addition to soluble molecular-mediated direct contact and signal transduction, leading to a variety of diseases, including thrombosis, in which exosomes plays an important role. The biological function of exosomes is to mediate intercellular communication by transferring critical biological substances, such as functional proteins, mRNAs, and microRNAs (miRNAs) (Gurunathan et al., 2019), to distal or adjacent recipient cells. The mechanisms of exosome formation can be divided into three stages: endosomal processing and formation of multivesicular bodies (MVBs), redirection from the lysosomal degradation pathway to the cell surface, and release.

In this review, we focus on the mechanisms by which exosomes influence VTE and their potential clinical applications.

## 2 Characterization of exosomes

**FIGURE 1:**
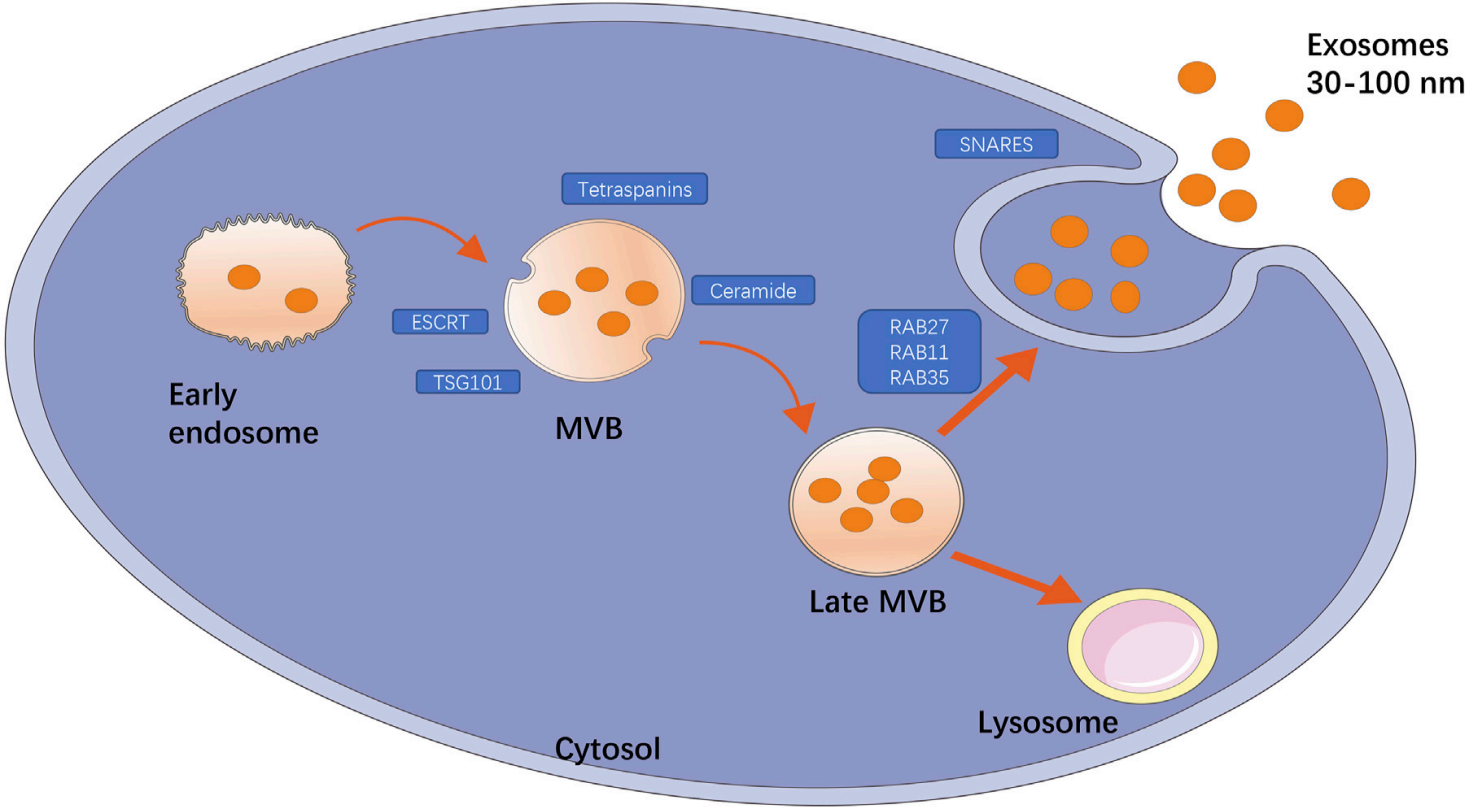
Title: The main mechanism of exosome production. The formation of exosomes is divided into three processes: endosomal processing formation of MVB; the intracellular transport of MVB that can be transported to lysosomes for degradation or to cell surface; the release of exosomes. Among them, some key factors play an important role, such as ESCRT, TSG101, ceramide, tetraspanins are involved in the formation of MVB, GTP enzymes of Rab family (Rab11, Rab27, Rab35) are involved in membrane transport, and SNARE complexes are involved in membrane fusion.

Exosomes are a special subtype of secretory cell-derived vesicles of endosomal origin that are 30–150 nm in diameter, and consist of a phospholipid bilayer embedded with proteins or small molecules, functional proteins, nucleic acids, and other metabolites. At present, the main mechanisms of exosome formation are believed to include both the endosomal sorting complex required for transport (ESCRT)-dependent and ESCRT-independent pathways. As shown in Figure 1, the exosome formation process can be divided into three stages: the first stage is endosomal processing and formation of MVBs, in which ESCRT(Raiborg and Stenmark, 2009), tumor susceptibility gene 101 protein (Nabhan et al., 2012), sphingomyelin, and ceramides (Trajkovic et al., 2008) play an important role. The second stage is the intracellular transport of MVBs. MVBs can be transported to lysosomes for degradation or to the cell surface, and this depends on interactions with the actin and microtubule cytoskeleton. The third stage is the release of exosomes, which results from the fusion of MVBs and cell membranes (Raposo et al., 1996). Exosomes are found in all body fluids, including plasma, urine, saliva, and breast milk (Zhang et al., 2015). More than 4000 proteins have been found in exosomes (Maas et al., 2017), including proteins involved in membrane transport and fusion such as small GTP enzymes of the Rab family (Rab11, Rab27, and Rab35), the SNARE complex (Cocucci and Meldolesi, 2015), integrins, flotillin, annexins, LAMP1, phospholipases, heat shock proteins, ESCRT-related proteins (e.g., ALIX, TSG101), and tetraspanins (CD9, CD63, CD81, CD82) (Hafiane and Daskalopoulou, 2018). In addition, exosomes are rich in phospholipids with long, saturated fatty acid acylated chains and important lipids such as cholesterol, sphingomyelin, and ceramides, the latter of which play an important role in vesicle germination (Trajkovic et al., 2008; Bissig and Gruenberg, 2014). In addition to functional proteins and lipids, exosomes also possess certain conserved glycosylation characteristics. Exosomes contain high levels of mannose, polylactosamine, α2–6-linked sialic acids, and complex N-linked glycans (Batista et al., 2011). Importantly, exosomes also contain a large number of RNA fragments, particularly miRNAs. Turchinovich et al. found a relationship between exosomes and parent cells by high-throughput RNA sequencing (R = 0.75), and a large number of miRNAs were detected only in exosomes, but not in apoptotic bodies and MVs, which supported the concept of exosomes enriched with specific RNA (Turchinovich et al., 2019). The type and content of biomolecules, such as lipids and metabolic micro molecules, carried by exosomes can directly reflect the cell type from which they are derived and the metabolic state the cells are in. Thus, exosomes not only contribute to a better understanding of cellular physiology and pathology, but also show great potential for transformation into clinical applications, ranging from diagnosis and prognosis to nucleic acid delivery and therapy. As a delivery system, exosomes cannot only protect bioactive proteins, lipids, RNAs (mRNAs, miRNAs, long non-coding RNAs), and DNA from degradation in the plasma, but also deliver exosome contents and convey their information to adjacent or distal cells, inducing functional responses and promoting physiological and pathological changes. Because exosomes contain large amounts of genetic material, they can also transmit genetic information that induces temporary or lasting modifications in recipient cells (Valadi et al., 2007; Tetta et al., 2013).

### 2.1 Isolation of exosomes

Exosomes are an attractive non-invasive diagnostic tool because they retain characteristic markers found in the cell of origin. At present, a variety of isolation and purification technologies for exosomes have been developed according to their biophysical properties, such as density, size, and shape, as well as their biochemical properties, such as surface antibodies and biofilms. However, in clinical practice, their use as biomarkers is hampered by technical challenges regarding the isolation, quantification, and identification of exosomes (Coumans et al., 2017; Ridger et al., 2017; Sluijter et al., 2018). To overcome these problems, the International Society of Thrombosis and Hemostasis, the European Society of Cardiology, and the International Extracellular Vesicle Society have provided a set of recommendations for the isolation and identification of exosomes (Coumans et al., 2017; Ridger et al., 2017; Sluijter et al., 2018; Thery et al., 2018; van der Pol et al., 2018). Exosomes can be isolated by ultracentrifugation, immunopurification, density gradient, commercial kits, and size-exclusion chromatography, among others. Of these, ultracentrifugation accounts for 56% (Zarovni et al., 2015), which is the gold standard for exosomes isolation (Coughlan et al., 2020). Although the isolation technology is relatively mature, the purification of exosomes still faces challenges. This is mainly due to the size of the vesicles and the difficulty of isolating samples from non-vesicle contaminants, such as protein aggregates, lipoproteins, and organelles (Zara et al., 2019). Taylor et al. believe that the shortcomings of the existing isolation methods (such as the presence of interferers in the separation products and the destruction of exosome structure) might lead to misleading downstream analysis of exosomes (Taylor and Shah, 2015). Hence, more precise separation techniques are needed.

### 2.2 Detection of exosomes

**FIGURE 2:**
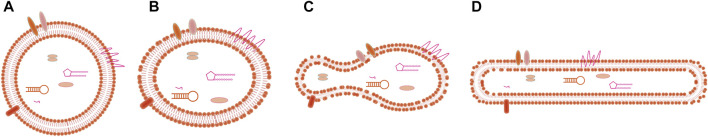
Title: Schematic of the morphology of exosomes. **(A)** Spherical shape, **(B)** Ellipsoid shape, **(C)** Cup-shape, **(D)** Tubular shape.

The most common methods to identify exosomes are scanning electron microscopy (SEM) and transmission electron microscopy (TEM). Exosomes from different biological sources are uneven in shape; for instance, exosomes may be spherical, ellipsoid, tubular, or cup-shaped, among others (Arraud et al., 2014; Hoog and Lotvall, 2015; Zabeo et al., 2017; Lasser et al., 2018), and schematic of the morphology of exosomes are shown in Figure 2. Sokolova et al. (Sokolova et al., 2011) characterized exosomes from three human cell types (HEK293T, ECFC, and MSC) by SEM, and found that all exosomes were spherical. However, the extracellular vesicles observed by Thery et al. (Thery et al., 2006) showed a twisted cup shape, although this may have been due to vesicle deformation caused by chemical fixation and dehydration during sample preparation. Whether the size of isolated and purified exosomes conforms to the specified particle size range is the key to identification. Dynamic light scattering (DLS) and nanoparticle tracking analysis (NTA) both use optical means to track the Brownian motion of nanoparticles in suspension and calculate the size distribution of exosomes to perform statistics (Shao et al., 2018). Atomic force microscopy (AFM) can also reflect the size of exosomes. In addition, western blotting and flow cytometry can be used to identify exosomes based on their protein composition.

## 3 Exosomes and thrombosis

Exosomes released by circulatory system cells (e.g., platelets, erythrocytes, leukocytes, and endothelial cells) are involved in the pathological mechanism of thrombosis. Thrombus formation is mediated directly by vascular and blood cells, but also by intercellular communication and connection via exosomes released by different types of cells involved in clotting.

The released exosomes selectively adhere to recipient cells and directly stimulate them, resulting in a series of pathophysiological processes (Del Conde et al., 2005). Studies have shown that arachidonic acid, PAF-like lipids, and lipoxygenase products transferred to recipient cells by exosomes play important roles in thrombosis regulation. Exosomes are rich in mRNAs/miRNAs. By selective cargo transfer of exosomes, recipient cells can possess both pro-inflammatory and anti-inflammatory properties (Alexander et al., 2015). Exosomes can deliver specific mRNAs/miRNAs that promote angiogenesis of resting endothelial cells when released from endothelial progenitor cells (Sun et al., 2019), and progenitor differentiation of functional megakaryocytes when released from mature megakaryocytes (Jiang et al., 2017).

**FIGURE 3:**
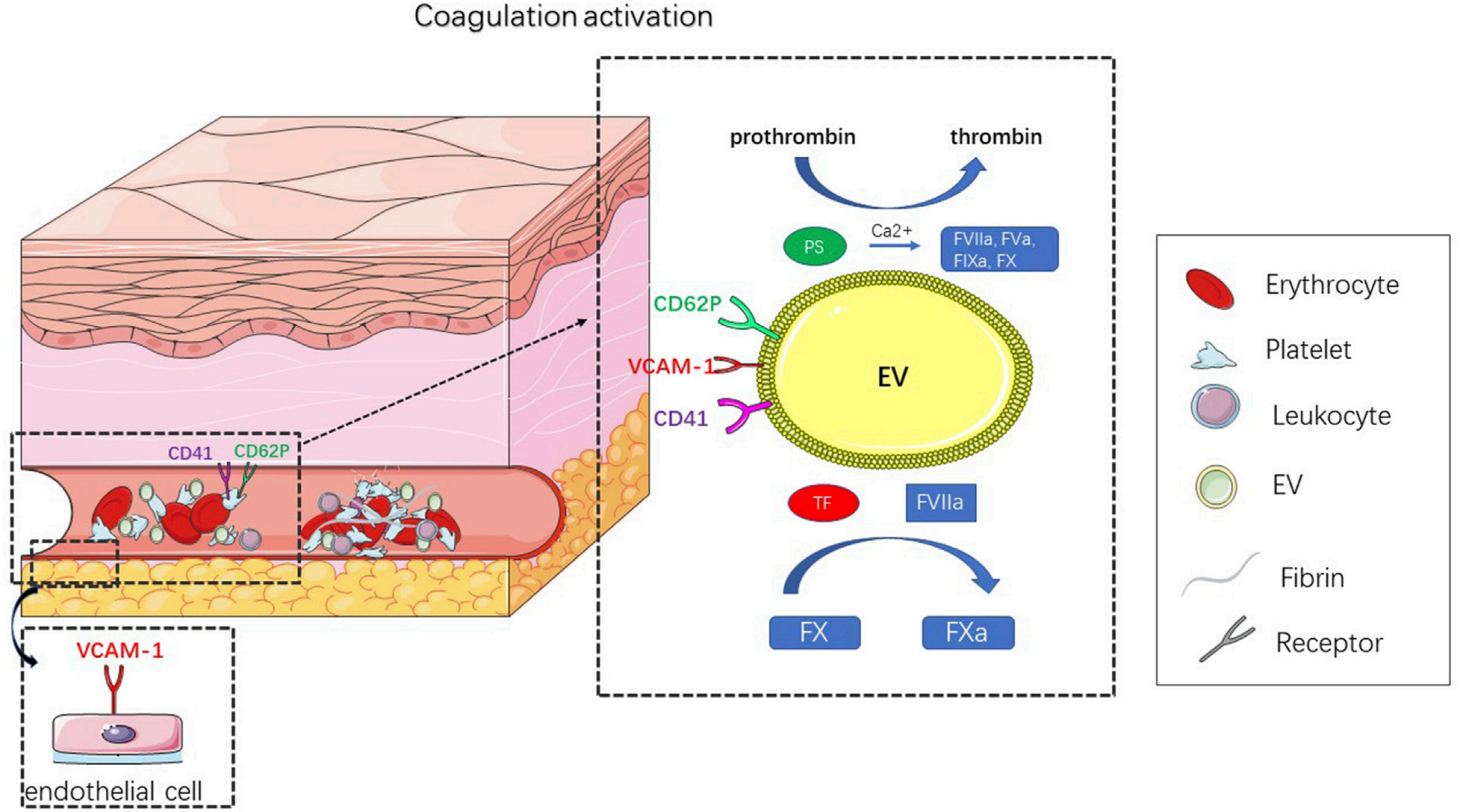
Title: Schematic diagram of EV leading to thrombosis. Thrombosis results from abnormal coagulation activation. In response to external stimulation, platelets, endothelial cells, erythrocyte and leukocyte can produce EV, that activate coagulation. The mechanism of coagulation depends on the expression of TF and PS. In the presence of calcium ions, these externalized PS can facilitate the assembly and activation of tendineae (factor VIIIa, IXa, X) and prothrombin (factor Va, Xa) complexes, thus promoting the formation of thrombin. TF is a key activator of the clotting cascade and can activate FVII, leading to the activation of FX, whose abnormal activation can result in thrombosis.

The formation of thrombi is mainly related to the expression of highly procoagulant proteins [e.g., tissue factor (TF)(Giesen et al., 1999)] and the exposure of negatively charged phospholipids (e.g., phosphatidylserine [PS](Reddy and Rand, 2020)) (Figure 3). EVs are thought to act as coagulants because the formation of EVs leads to the externalization of anionic phospholipids (especially PS). In the presence of calcium ions, externalized PS can facilitate the assembly and activation of tendineae (factor VIIIa, IXa, and X) and prothrombin (factor Va and Xa) (Heemskerk et al., 2002) complexes, thus promoting the formation of thrombin (Owens and Mackman, 2011). The expression of TF further enhances the procoagulant activity of EVs. TF is a powerful promoter of the exogenous clotting pathway and is a key activator of the clotting cascade and can activate FVII, causing hemostasis after vascular injury; abnormal activation of TF can lead to thrombosis (Mackman, 2009). At the site of vascular injury, TF produced by the vascular wall plays a major role in the initial stage, while blood-derived TF participates in the transmission stage of thrombosis (Hathcock and Nemerson, 2004; Camera et al., 2015). In healthy people, the number of TF-positive EVs is very small, but under pathological conditions, the number of TF-positive EVs increases rapidly (Schorey and Harding, 2016). In addition, TF activity is affected by posttranslational modifications, such as glycosylation. Batista et al. (Batista et al., 2011) showed that MV originates from a conserved part of the cell membrane and has a unique carbohydrate composition. High mannose, complex N-linked glycans, α-2,6 sialic acid and poly-N-acetyllactosamine epitopes are enriched on the microvesicles, and these carbohydrate epitopes can guide TF to be directed to the surface of MV membrane and trigger coagulation cascade.

Circulating cells (platelets, erythrocytes, leukocytes, and endothelial cells) are involved in the formation of thrombi. Next, we will discuss how these circulating cells lead to thrombosis.

### 3.1 Platelet and megakaryocyte-derived vesicles

Platelet extracellular vesicles are the most abundant EV in the human body and include two types: microvesicles, commonly known as platelet-derived microparticles (PMPs), and exosomes (Boilard et al., 2015). PMPs can be detected by platelet representative markers, such as CD41 (integrin αIIb) and CD42b (glycoprotein, GPIB), and platelet-specific activation markers, such as CD62p (p-selectin) and fibrinogen receptor integrin αIIbβ3. Platelet-derived exosomes are derived from MVBs and α particles (Heijnen et al., 1999). In addition to typical exosomal markers, platelet exosomes are also rich in CD41 and specific miRNAs, including miR-21, miR-223, and miR-339 (Tan et al., 2016; Li J. et al., 2017; Guo et al., 2017).

A variety of agonists can activate platelets to release extracellular vesicles, including adenosine diphosphate (ADP), thrombin, collagen, and the thrombin receptor agonist peptide SFLLRN (TRAP) (Thiagarajan and Tait, 1991; Brisson et al., 2017; Gasecka et al., 2020). Platelet-derived extracellular vesicles induce coagulation mechanisms that depend on the expression of TF and PS. Studies have shown that EVs isolated from healthy human platelets support *in vitro* coagulation through a TF-independent pathway involving factors FXII, XI, IX, and VIII(Mooberry et al., 2016). PS alone can lower the activation threshold of coagulation via stronger stimulation and can promote the interaction between different circulating cells involved in thrombosis. In addition, platelet-derived vesicles can rapidly adhere to monocytes via P-selectin, resulting in the transfer of platelet GpIbα to monocytes and their interaction with vWF(Chimen et al., 2020).

Notably, platelet-derived EVs bind not only to annexin V and lactadherin, but also to protein S, supporting the anticoagulant activity of activated protein C (Koshiar et al., 2014; Somajo et al., 2014). In addition, exosomes released from activated platelets reduce CD36 in platelets and macrophages via ubiquitination and proteases (Steppich et al., 2005). Platelet-derived exosomes carrying miR-320 can promote endothelial cell mobility, reduce inflammation and thrombosis, and reduce ICAM-1 expression in endothelial cells (Gidlof et al., 2013). Platelet-derived exosomes carrying miR-223 inhibit ICAM-1 expression during inflammation by regulating the NF-κB and MAPK pathways (Li J. et al., 2017).

### 3.2 Endothelial-derived vesicles

Endothelial-derived EVs have been identified as biomarkers for endothelial dysfunction (Dickhout and Koenen, 2018). Circulating EC-MVs have endothelial cell markers: CD31, CD105, CD144, CD62E, and vascular cell adhesion molecule-1 (VCAM-1) (Dignat-George and Boulanger, 2011; Hromada et al., 2017). Endothelial cells secrete exosomes rich in specific miRNAs, such as miR-214, miR -210, miR -126, and miR -146a (Halkein et al., 2013; van Balkom et al., 2013; Ong et al., 2014).

In general, endothelial-derived vesicles has coagulant and fibrinolytic activity. The final result of these activities depends on achieving a molecular balance between coagulant and fibrinogen. This balance is influenced by the internal and external environment and the factors that stimulate it.

Endothelial-derived vesicles showed increased surface expression of PS and TF, which partly explains their procoagulant activity. Negatively charged PS causes vesicles to bind and activate clotting factors (Owens and Mackman, 2011). TF is a promoter of exogenous clotting pathways (Schorey and Harding, 2016). Circulating TF- and PS-positive endothelial-derived EVs increase the risk of venous thrombosis. Similarly, EVs from endothelial cells can activate hemostasis and clotting by modulating inflammatory responses. EC-EVs can stimulate the production and release of inflammatory cytokines, the expression of adhesion molecules on endothelial cells and white blood cells, and activate coagulation and hemostasis (Buesing et al., 2011; Eyre et al., 2011; Robbins and Morelli, 2014). For example, MVs produced by activated endothelial cells may spread procoagulant and pro-inflammatory potential by binding to adhesion molecules (e.g., ICAM1) on immune cells and transferring bioactive TF *in vitro*, such as THP1 monocytes (Sabatier et al., 2002). Thrombin produced during the inflammatory response activates endothelial cells via PAR-1, a G protein-coupled receptor, which stimulates EV release and the expression of several cytokines, chemokines, and receptors, including IL-8, IL-1Ra, and IL-1, further promoting EV release (Markiewicz et al., 2013).

Interestingly, EC-EVs also express partial anticoagulation ability. On one hand, endodermal MPs contain active TF pathway inhibitor (TFPI), which can offset TF activity and prevent abnormal activation and expression of TF (Steppich et al., 2005). Conversely, the presence of endothelial protein C receptor (EPCR, CD201) on the surface of vascular endothelial cells also leads to anticoagulant activity in vessels (Brown et al., 2011). It has been shown that MVs isolated by stimulation of endothelial cells with TNFα can detect elevated levels of urokinase-type plasminogen activated receptor (uPAR) on their surface, leading to plasminogen production (Lacroix et al., 2007), which can lead to thrombolysis. Furthermore, exosomes derived from endothelial progenitor cells carrying miR-126 can significantly promote thrombolysis. Exosomes carrying miR-126 enhance endothelial progenitor cell (EPC) migration and angiogenesis (Sun et al., 2019).

### 3.3 Leukocyte-derived vesicles

Immune cells, including T and B cells, dendritic cells, monocytes, macrophages, mast cells, natural killer cells, and granulocytes all release EVs. Most of the extracellular vesicles released by these immune cells contain inflammatory cytokines, intracellular cell adhesion molecule-1 (ICAM-1), P-selectin glycoprotein ligand-1 (PSGL-1), tissue factor (TF), complement receptor 3 (C3), metalloproteinases, tRNAs, mRNAs, miRNAs, and long non-coding RNAs(Nolan et al., 2008; Pluskota et al., 2008; Wang et al., 2011; Liu et al., 2016; Liu et al., 2019). Monocyte-derived EVs express CD11b, CD14, CD64, and CD142, whereas neutrophil-derived EVs express CD35, CD66b, and myeloperoxidase. Notably, MVs released by megakaryocytes share the expression of CD41, CD42b, GPVI, and other typical markers with PMPs(Flaumenhaft et al., 2009), but do not share P-selectin and other platelet activation marker expression. Under external stimulation, monocyte-derived exosomes are enriched with miR-222, miR -155, miR -146a, miR -146b, and miR -125A-5p (Dalvi et al., 2017).

Leukocyte-derived vesicles usually develop following inflammatory responses that involve overexpression of TF in the blood and/or endothelial cells, which promotes thrombin production and participates in the early stages of thrombosis, especially in monocytes and neutrophils (von Bruhl et al., 2012). For example, during an immune response to bacterial infection, activated platelets bind to neutrophils to stimulate the release of arachidonic acid-containing exosomes. These exosomes are then transmitted to platelets, supporting the production of pro-inflammatory and pro-aggregation mediators (Rossaint et al., 2016). A hemostatic test showed that monocyte-derived EVs had the highest ability to induce coagulation via the TF pathway, whereas platelet- or erythrocyte-derived EVs activated coagulation only via the contact pathway (Lipets et al., 2020). Previous studies have shown that the majority of EVs expressing TF are derived from monocytes, and compared with platelets, monocyte-derived EVs have stronger procoagulant activity (Aleman et al., 2011; Van Der Meijden et al., 2012). Conversely, monocyte EVs can promote fibrin formation and increase the density of the fibrin network, thus promoting blood clot stability (Aleman et al., 2011).

### 3.4 Erythrocyte-derived vesicles

In addition to platelets, endothelial cells, and leukocytes, erythrocytes (red blood cells), the most abundant cells in the circulation, also release EVs. The release of vesicles by erythrocytes can occur following an intracellular calcium influx and calcium independence from oxidative stress (Cloos et al., 2020), osmotic shock, or activation of protein kinase C (PKC) (Nguyen et al., 2016). Specific markers of erythrocyte-derived MVs include membrane proteins band 3, actin, hemoglobin A, CD55, CD59, iron, annexin A1, annexin A2, and GLUT1. Erythrocyte-derived exosomes are formed during reticulocyte maturation or in stored erythrocyte units, they express CD235a and CD63 (Danesh et al., 2014).

Most erythrocyte derived-EVs express PS on their surface, which can bind lactadherin and annexin V to assemble tenase and prothrombinase complexes, thus supporting thrombin production (Koshiar et al., 2014). Erythrocyte-derived MPs can increase the expression of monocyte TF and promote platelet–monocyte aggregation in healthy humans (Fischer et al., 2017). Similar to platelets, erythrocyte-derived EVs can trigger thrombin production via factor XIIa and enhance contact factor pathway-mediated coagulation (Van Der Meijden et al., 2012), they can also initiate clotting by directly activating factor XII or pre-kallikrein, leading to factor IX activation (Noubouossie et al., 2020). In addition, erythrocyte derived-exosomes can stimulate monocytes to produce TNFα and enhance T cell proliferation to promote inflammation.

## 4 Clinical applications

The biological function of exosomes is to bind functional proteins, lipids, and RNA from parental cells and deliver these factors to recipient cells or tissues to induce a series of pathophysiological reactions. In the process of thrombosis, understanding the function of exosomes may aid diagnosis, treatment, therapeutic monitoring, and prognosis. The analysis of counts, surface marker expression, protein, and miRNA cargo in exosomes may be useful. Therefore, exosomes may be used as non-invasive diagnostic tools.

Elevated levels of circulating exosomes are primarily associated with venous thrombosis. For example, the levels of miR-223, miR-339, and miR-21, which are associated with platelet activation in plasma exosomes, were elevated prior to thrombosis (Tan et al., 2016). It has been reported that APS-exosomes inhibit the migration and tubular formation of human umbilical vein endothelial cells (HUVECs) in antiphospholipid syndrome (APS). Apoprotein H (APOH) may be the core protein in this process. APOH-exosomes play a role in promoting thrombogenesis by phosphorylating the extracellular signal-regulated kinase pathway (Tan et al., 2021). Thus, monitoring the changes in these exosomes, miRNAs, and proteins could predict thrombosis.

Another clinical application of exosomes is as a drug delivery system. Studies have shown that miR-126 can inhibit the expression of target gene, *PIK3R2*, activate the PI3K/Akt signaling pathway, enhance the recruitment of EPCs in venous thrombosis, and improve the migration and angiogenesis of EPCs(Meng et al., 2015). Translocation of miR-126 into EPCS-released exosomes by electroporation inhibited the expression of EPC protocadherin-7 and promoted deep vein thrombosis resolution and recanalization (Sun et al., 2019). Platelet-derived exosomes carrying miR-320 can promote endothelial cell mobility, reduce inflammation and thrombosis, and reduce ICAM-1 expression in endothelial cells (Gidlof et al., 2013). Platelet-derived exosomes carrying miR-223 inhibit ICAM-1 expression during inflammation by regulating the NF-κB and MAPK pathways (Li J. et al., 2017).

In general, exosomes will undoubtedly be a future tool for DVT detection and treatment. On the one hand, the composition of blood is very complex, and some biological molecules such as proteins secreted by cells are diluted in the blood, so it is not easy to detect in the early stages or at low levels, but exosomes are wrapped in membranes and can be isolated intact, and the molecules they carry are not diluted (Li A. C. et al., 2017; Li W. et al., 2017; Hsu et al., 2022). Thrombus related proteins, lipids, mRNA, non-coding RNA and DNA in exosomes can be used to detect thrombus. On the other hand, exosomes are widely found in body fluids and are easy to obtain and detect. Exosomes are small in size and have a strong ability to pass through barriers. Compared with synthetic nanoparticles, exosomes are biocompatible and biodegradable (Witwer and Wolfram, 2021), so they have higher affinity and lower antigen. The intracellular adhesion and internalization of exosomes are 10 times higher than liposomes of the same size (Witwer and Wolfram, 2021), indicating that exosomes are more targeted to disease. In addition, the lipid bilayer structure of exosomes protects their contents from degradation by enzymes circulating in the blood. Some thrombolytic drugs can directly target thrombi by embedding exosomes, reducing the loss of drug degradation.

However, as a new detection and treatment tool, exosomes have some problems to be solved in clinical application.1. The standardization and quality control of exosomes will have to take industrial production and regulatory standards into account before they can be used in human therapy, as well as the manufacturing costs of mass production;2. Because the contents of exosomes are different from the type and state of parental cells, the selection of the type of exosomes provided will be critical and should be screened according to the type of recipient cell and target molecule;3. A large number of studies on the correlation of exosome-mediated signal transduction in the occurrence and development of DVT are needed to prove its unique therapeutic target.

## 5 Conclusion

Exosomes are nanoscale extracellular vesicles secreted by various cells that carry small molecules, such as proteins and nucleic acids, that participate in intercellular communication. Several studies have focused on the effects of exosomes on coagulation, inflammation, angiogenesis, apoptosis, differentiation, and cell migration. In this paper, we discussed the influence of exosome on thrombosis. The literature shows that the prethrombotic characteristics mainly depend on the expression of PS and TF. In addition, the presence of other procoagulants (e.g., PSGL-1 and GPIIb/IIIa), intercellular interactions, and activation of signaling pathways may also play a significant role.

Interestingly, thrombosis often results in changes in the number of extracellular vesicles and their protein, RNA, or miRNA contents; therefore, exosomes could be used as biomarkers not only to diagnose thrombosis but to reflect the severity and prognosis of the disease. Extracellular vesicles are found in all body fluids, including plasma, urine, saliva, and breast milk; hence, their measurement could provide an alternative to other invasive biopsy tools. In addition, exosomes can serve as drug delivery vehicles for the distribution of therapeutic drugs to target tissues in the event of disease and injury, given their intercellular communication capabilities. However, the development of these applications requires that intact exosomes can be isolated without impurities. The challenge for future research is to standardize the isolation, identification, and analysis of exosomes.

In conclusion, research on the function and molecular mechanisms of exosomes is still in its infancy, and further study is required. Under pathophysiological conditions, researchers should determine 1. Changes in effective or key molecules in exosomes, 2. the interaction between effective molecules, 3. the process of effective molecules entering exosomes, 4. the deep mechanism of exosome production and release, 5. the regulation mechanism of exosome directional transport, and 6. the mechanisms by which target cells recruit and activate exosomes. Furthermore, it is not clear whether effective molecules have different functions when they are located and distributed differently in time and space between cells and exosomes. These are all things we need to determine in future exosome research.

## Abbreviations

MVB, multivesicular bodies; ESCRT, endosomal sorting complex required for transport; TSG101, tumor susceptibility gene 101.
